# Reviving vacuum-dried encapsulated ram spermatozoa via ICSI after 2 years of storage

**DOI:** 10.3389/fvets.2023.1270266

**Published:** 2023-11-30

**Authors:** Luca Palazzese, Federica Turri, Debora Agata Anzalone, Joseph Saragusty, Jacques Bonnet, Marthe Colotte, Sophie Tuffet, Flavia Pizzi, Alessia Luciani, Kazutsugu Matsukawa, Marta Czernik, Pasqualino Loi

**Affiliations:** ^1^Institute of Genetics and Animal Biotechnology of the Polish Academy of Sciences, Warsaw, Poland; ^2^Institute of Agricultural Biology and Biotechnology (IBBA), National Research Council (CNR), Lodi, Italy; ^3^Department of Veterinary Medicine, University of Teramo, Teramo, Italy; ^4^Laboratoire de Recherche et Développement, Imagene Company, Pessac, France; ^5^Institut Bergonié, INSERM, Université de Bordeaux, Bordeaux, France; ^6^Plateforme de Production, Imagene, Genopole, Evry, France; ^7^Faculty of Agriculture and Marine Science, Kochi University, Kochi, Japan

**Keywords:** encapsulation, lipidomics, polyunsaturated fatty acids, ICSI, ovine

## Abstract

**Introduction:**

Freeze-drying techniques give alternative preservation mammalian spermatozoa without liquid nitrogen. However, most of the work has been conducted in the laboratory mouse, while little information has been gathered on large animals that could also benefit from this kind of storage.

**Methods:**

This work adapted a technique known as vacuum-drying encapsulation (VDE), originally developed for nucleic acid conservation in anhydrous state, to ram spermatozoa, and compared it to canonical lyophilization (FD), testing long-term storage at room temperature (RT) and 4°C.

**Results and discussion:**

The results demonstrated better structural stability, namely lipid composition and DNA integrity, in VDE spermatozoa than FD ones, with outcomes at RT storage comparable to 4°C. Likewise, in VDE the embryonic development was higher than in FD samples (12.8% vs. 8.7%, *p* < 0.001, respectively). Our findings indicated that in large mammals, it is important to consider dehydration-related changes in sperm polyunsaturated fatty acids coupled with DNA alterations, given their crucial role in embryonic development.

## Introduction

Cryobiobanking in liquid nitrogen is a universally-applied and well-mastered methodology, but it is costly, energy-expensive, and has a sizeable carbon footprint (1), thus restricted to wealthy countries that have the facilities to produce liquid nitrogen and deliver uninterrupted energy supply. An alternative way for biobanking was first successfully presented in 1998 when Wakayama and Yanagimachi (2) produced offspring using freeze-dry mouse spermatozoa delivered by intracytoplasmic sperm injection (ICSI). Initially treated with indifference by the scientific community, dry storage of male gametes started to gain attention when repeated in other species. We believe that developing alternative and low-cost storage procedures is highly desirable for two main reasons. First, every undertaking that cuts the carbon footprint, like establishing and maintaining dry biobanks with storage at room temperature, is welcome and in line with global policies. Second, semen storage in the anhydrous state, besides being user-friendly and low-cost, simplifies transportation enormously; this is crucial in the era of space travel, as already demonstrated by dry mouse spermatozoa being able to retain fertilizing capacity following exposure to cosmic radiation (3).

However, while most knowledge in the field was gained on laboratory animals such as rabbits (4), rats (5), and hamsters (6), very little research was done in farm animals such as pigs (7), cattle (8), horses (9), and sheep (10).

It is generally suggested to store vials with freeze-dried spermatozoa at sub-zero temperatures (−20°C, or even better, at −80°C), especially if long-term storage is intended (11), distancing the technique from the ideal and decisive energy-cheap room temperature storage. Full sustainable shipping and storage of spermatozoa in the dry state were demonstrated only in the ovine (12) and murine model (13). Efforts should concentrate on large mammal models for future applications in farm animals and, subsequently, in human assisted reproduction. The current state-of-the-art in drying male gametes is ill-defined, very fluid, and uncertain in terms of whether freezing should be applied, the methods chosen for water removal, and the rehydration approach. A recent review highlighted the availability of 10 other methods for water subtraction; however, only canonical lyophilization has been extensively explored, with the others receiving only marginal attention (14). This is a major shortcoming. If we observe how anhydrobiotic organisms survive desiccation, freezing and water sublimation should have received more attention.

Following an exhaustive literature review, we found a nucleic acid preservation method for long-term storage in the anhydrous state at room temperature, vacuum-drying followed by encapsulation (15), and decided to apply it to desiccating ram spermatozoa.

This study aimed to explore this alternative water extraction and packaging strategy to preserve structural integrity at the membrane and DNA levels and, consequently, the sperm fertilizing capacity using ram spermatozoa as a model. Furthermore, the effects of long-term storage temperature were analyzed.

## Materials and methods

### Ethics approval

The animal experiment (semen collection) has been approved by the Italian Ministry of Health (No. 200/2017-PR) based on the research description prepared by the ethics committee of the Istituto Zooprofilattico Sperimentale di Teramo (Prot. 944F0.1 del 04/11/2016). All methods were performed following the relevant guidelines and regulations of the Italian Minister of Health.

### Chemicals

Unless otherwise stated, all materials used were purchased from Sigma Aldrich (St Louis, MO, USA).

### Semen collection

Semen of a fertile Sardinian ram whose reproductive ability was previously confirmed (10, 16) was collected using an artificial vagina filled with warm water (40–44°C) and connected to a 15-mL sterile tube. Sperm motility was evaluated using a stereomicroscope immediately after collection. The sample was diluted 1:1 with Basic Medium (300 mM TRIS base, 105 mM citric acid, 82 mM fructose, 150,000 IU penicillin G, 2 mM streptomycin in 67.2 mL bi-distilled water) and transported to the laboratory in a transportable incubator (INC-RB1 Biotherm, Cryologic, Blackburn, Australia) at 32–35°C.

**Figure 1:**
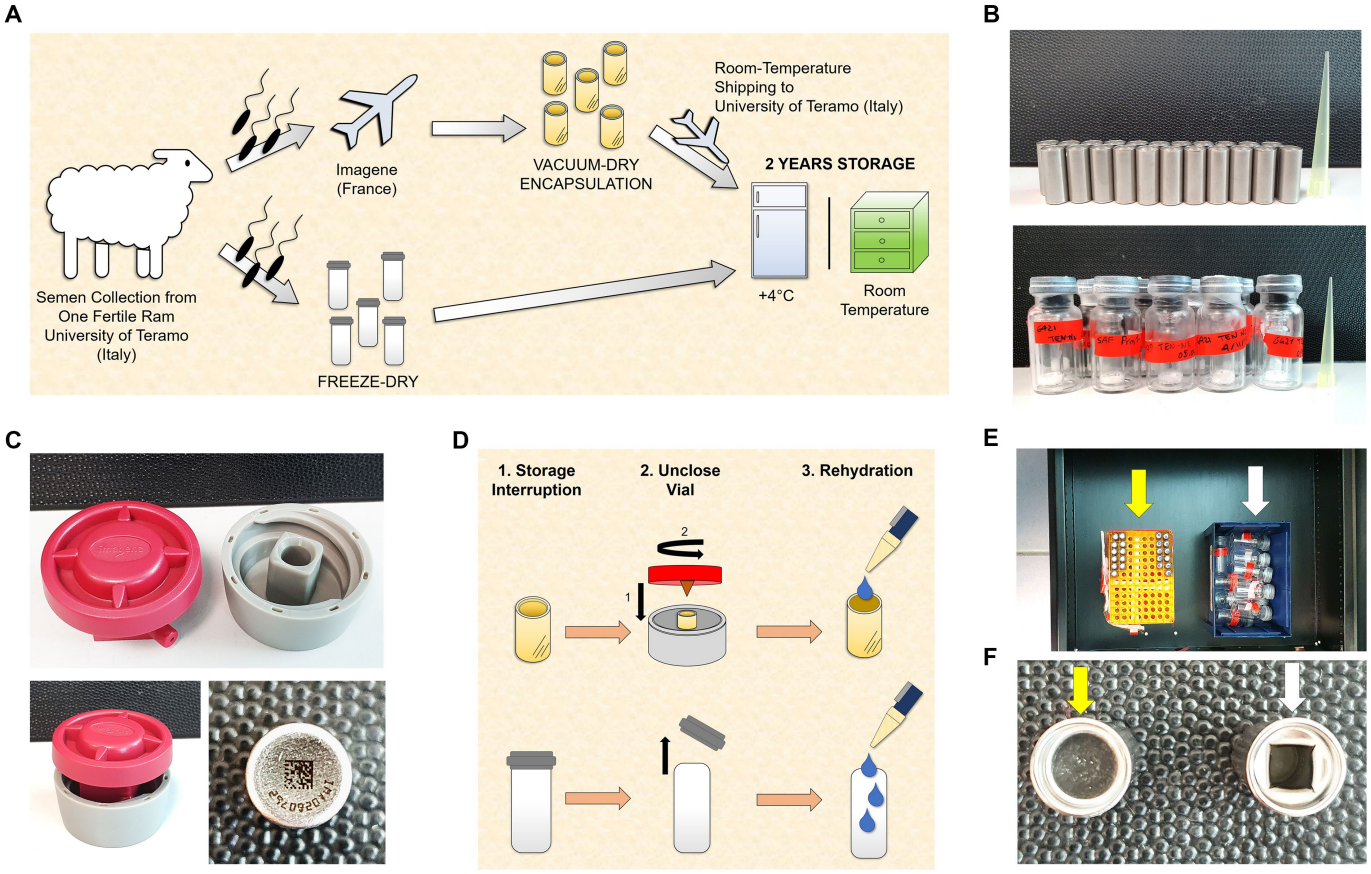
Experimental design. **(A)** Semen collection was carried out at the University of Teramo (Italy). An aliquot was freeze-dried (FD) immediately, and another aliquot was sent to Imagene (France) to perform the vacuum drying followed by encapsulation (VDE). The VDE spermatozoa were returned to the University of Teramo at room temperature and stored with the FD samples for 2 years at 4°C (4C) and in a drawer at room temperature (RT). **(B)** Stainless steel capsules containing the VDE spermatozoa sealed by laser under an inert gas atmosphere in the top panel. Glass vials [small (Ø 8 mm) in large (Ø 20 mm)] with the FD spermatozoa sealed under vacuum in the bottom panel. **(C)** The *ad-hoc* shellOpener was developed to open the VDE capsules (top and bottom left pictures). The sample number and QR code on the bottom of the capsule label the VDE samples (bottom right image). **(D)** The FD and VDE samples, stored for 2 years, were opened and rehydrated after two years of storage. VDE minicapsules (upper row) were opened using the *ad-hoc* shellOpener. **(E)** FD (white arrow) and VDE (yellow arrow) spermatozoa stored for 2 years in a drawer. **(F)** Top view of a sealed VDE (yellow arrow) and opened (white arrow) minicapsule.

Upon arrival, the ejaculate was split in two and was processed at two institutions by two drying techniques (FD and VDE; Figure 1 and the following subsections).

All experiments described herein were performed using a single ejaculate to reduce variability and produce the most objective observations of the tested variables.

### Sperm cryopreservation

Semen cryopreservation was conducted following the established protocol outlined in (16). In brief, the freezing medium was prepared as follows: a Basic Medium was created by dissolving 2.42 g of TRIS base, 1.36 g of citric acid, 1.00 g of fructose, 100,000 IU of penicillin G, and 0.1 g of streptomycin in 67.20 mL of bi-distilled water, with the pH adjusted to a range of 6.7–6.8. Next, the Basic Medium was divided into two equal volumes, resulting in Medium A (maintained at 30°C) and Medium B (kept at 4°C). Medium A contained 20% egg yolk and 12.8% ddH2O, while Medium B contained 20% egg yolk and 12.8% glycerol. An equal volume of each of these two mediums was added to the ejaculate, achieving a final concentration of 400 × 10^6^ spermatozoa/ml. The process involved adding Medium A (at 30°C) to the ejaculate, which was then maintained at 4°C for a duration of 2 h. Subsequently, Medium B (at 4°C) was introduced and maintained for an additional 2 h at the same temperature. Throughout this process, tubes were agitated every 30 min. Finally, 250 μL straws were filled with the semen and allowed to equilibrate on liquid nitrogen vapors for 20 min. Following this, the straws were sealed and immediately submerged in liquid nitrogen.

### Sperm drying and samples storage

#### Sperm freeze-drying

Sperm FD was performed at the Faculty of Veterinary Medicine, University of Teramo (Italy), as previously described (10). Briefly, on arrival at the laboratory, the motile component was isolated by swim-up. Ejaculated semen (20 μL) was placed at the bottom of a 15-mL sterile tube containing 1.5 mL Basic Medium and left inclined at 45° for 20 min at 37°C. Subsequently, the top 750 μL containing the after swim-up spermatozoa was collected, and the concentration was corrected to 20 × 10^6^ spermatozoa/mL in the lyophilization medium (1 mL 0.5 M TRIS, 5 mL 0.5 M EGTA, 2.5 mL 1 M NaCl, all dissolved in bi-distilled water). Aliquots of 100 μL were placed in glass vials (Ø 8 mm), loaded into Mister Frosty (Thermo Fisher Scientific, Waltham, MA, USA), and progressively cooled to −50°C at a cooling rate of −1°C/min.

After freezing, the glass vials with the frozen samples were inserted into larger glass vials (Ø 20 mm), partially covered with stoppers, and placed inside the freeze-drying apparatus (VirTis BenchTop 2.0, SP Scientific, Gardiner, NY, USA) with the condenser temperature set at −58°C and the freeze-drying chamber at −12°C. As soon as the vials were placed in the lyophilizer, the vacuum pump was started, and drying proceeded for 16 h. The vials were sealed under a vacuum (pressure of 15 μbar). The freeze-drying chamber’s temperature reached −30°C.

#### Sperm vacuum drying encapsulation

The spermatozoa, suspended in the Basic Medium, were transferred at 4°C to Imagene (France). Upon arrival, the semen’s motility was assessed, yielding a motility rate of 80%. Subsequently, the tubes were centrifuged, and the pellet was resuspended in the lyophilization medium and the concentration was corrected to 20 × 10^6^ spermatozoa/mL. Aliquots (50 μL) were placed in stainless steel minicapsules and dried under vacuum for 55 min in an evapo-concentrator (HT4, Genevac, Ipswich, UK). The minicapsules were then transferred into a glove box and maintained for 72 h under an anoxic and anhydrous argon/helium atmosphere for further desiccation. At the end of the process, the minicapsules were capped with stainless steel caps and sealed by laser welding. Finally, the minicapsules were checked for leakage by mass spectrometry.

#### Storage condition and international room-temperature sample shipping

The ready minicapsules were shipped from France to Italy (University of Teramo) at room temperature. All samples (freeze-dried and vacuum-dried) were stored for 2 years, 30 (*n* = 15 each) at 4°C in the dark (FD_4C and VDE_4C, respectively) and 30 (*n* = 15 each) at room temperature (RT) in the dark (FD_RT and VDE_RT, respectively). The samples stored at RT were kept in a controlled environment with a temperature range between 20 and 25°C. At the end of the 2-years storage, the samples were used for *in vitro* fertilization by ICSI and flow-cytometric and lipidomic analyzes as described below.

### Opening and resuspension of the FD vials and VDE minicapsules

The VDE minicapsules were opened using an *ad hoc* shellOpener (Figures 1C,D,F). The minicapsules were placed in the special housing of the shellOpener, and the shellOpener lid was screwed, ensuring that the tip on the inside of the lid punched a hole in the minicapsules. For the FD vials, the rubber stopper was removed. All samples were rehydrated with bi-distilled water: 100 μL for FD samples and 50 μL for VDE samples. All samples were rehydrated within controlled environments, maintaining a temperature range between 20 and 25°C.

### Spermatozoa quality evaluation

#### Live-dead staining

After rehydration, samples were incubated with 0.1 μg/mL Hoechst 33342 (to mark all nuclei) and 5 μg/mL PI (to detect spermatozoa with damaged membrane) in PBS for 5 min at RT. Subsequently, a drop (15 μL) was placed on the slide, covered with a coverslip, and observed on an epifluorescence microscope (Eclipse E-600, Nikon, Tokyo, Japan).

#### Flow cytometry analysis

Measurements were recorded using a Guava EasyCyte 5HT microcapillary flow cytometer (Merck KGaA, Darmstadt, Germany) with fluorescence probes excited by a 20-mW argon ion laser (488 nm). Forward-scatter vs. side-scatter plots were used to separate the spermatozoa from debris by excluding non-sperm events from further analysis. Fluorescence detection was made with three photomultiplier tube detectors: FL-1 (green: 525/30 nm), FL-2 (yellow/orange: 586/26 nm), and FL-3 (red: 690/50 nm). Calibration was made using standard beads (Guava Easy Check Kit, Merck Millipore). We analyzed 5,000 sperm events per sample at a flow rate of 200 cells/s. Compensation for spectra overlap between fluorochromes was set according to the procedures outlined by (17). Data were acquired and analyzed using CytoSoft and EasyCompDNA software (Merck KGaA), respectively.

#### Acrosomal membrane integrity

Fluorescein isothiocyanate-conjugated peanut agglutinin (FITC-PNA) and Propidium Iodide (PI) at appropriate concentrations and 2.0 × 10^5^/mL rehydrated spermatozoa were added to Easy Buffer (IMV Technologies, l’Aigle, France). FITC-PNA labeled damaged acrosomes green, whereas PI-stained spermatozoa with damaged membrane red. Samples were incubated for 45 min at 37°C in the dark following the manufacturer’s protocol. Three replicates per sample were performed. After gating out non-DNA-containing particles, two populations were detected on the FL1/FL-3 dot plot, spermatozoa with damaged membranes with intact or damaged acrosomes.

#### Sperm DNA fragmentation

Sperm Chromatin Structure Assay (SCSA) (18) was used as previously described (10). Samples were stained with acridine orange, a fluorochrome that turns from red to green depending on the degree of chromatin compaction, to distinguish between denatured, single-strand, and double-strand DNA regions. The rehydrated FD and VDE sperm concentration was corrected to 20 × 10^6^ spermatozoa/mL. Twenty-five μL of the rehydrated sperm suspension was diluted in 195.75 μL TNE buffer [0.01 M Tris–HCl, 0.15 M NaCl, 1 mM ethylenediaminetetraacetic acid (EDTA), pH 7.4] and then added into 1,200 μL of an acidic solution (0.1% Triton X-100, 0.15 M NaCl, 0.08 M HCl; pH 1.2). After 30 s, the cells were stained with 1.2 mL acridine orange solution (6 μg/mL in 0.1 M citric acid, 0.2 M Na_2_HPO_4_, 1 mM EDTA, 0.15 M NaCl; pH 6.0). After 2.5 min, two replicates per sample were read on the flow cytometer.

Data were acquired and analyzed using cytoSoft and EasyCompDNA software, respectively (Merck KGaA). The DNA damage types were quantified with the following parameters: %DFI, DNA fragmentation index, the extent of DNA denaturation, i.e., the percentage of spermatozoa outside the main sperm population (those with fragmented DNA); %HG, percentage of spermatozoa with high green fluorescence, i.e., the immature cells part of DFI/cells with “high fragmented DNA.”

#### Glycerophospholipid extraction and analysis

Fresh refrigerated and dried spermatozoa of the four experimental groups (FD_4C, FD_RT, VDE_4C, VDE_RT) were analyzed in triplicate by gas chromatography (GC) with a flame ionization detection (FID) system using Agilent 7890B (Agilent Technologies, Milan, Italy) chromatographic system equipped with a Split/Splitless injector, FID system, and a 60 m × 0.25 mm × 0.25 μm DB-23 column.

Fresh chilled (250-μL) and rehydrated (100-μL) samples were centrifuged at 14,000 rpm at 4°C for 15 min. The pellets were transferred to an LNG-R1 robotic unit. The steps performed by the robotic unit were: (a) lipid extraction with 2:1 organic chloroform phase to methanol, collection, anhydrification with anhydrous sodium sulfate in vials, and vacuum-drying a Genevac evaporator; (b) treatment of the samples with 2 mL 0.5 M methanolic potash, stirring for 15 min at RT, adding 0.5 mL H_2_O, and extracting the methyl ester fatty acids with *n*-hexane. The extract was carefully mixed with 15 μL hexane, transferred into amber vials, positioned in the GC autosampler (Agilent 7693A), and processed through the analysis sequence. A wash sample (containing only n-hexane) was injected after running one or two samples and at the end of the sequence. The relative quantitative percentages of the fatty acids were calculated automatically by the software (Agilent OpenLab CDS ChemStation Edition 5.03; driver version 1.03). The software allows to express the results also as absolute concentrations in mg/mL.

### Oocyte recovery and *in vitro* maturation

Sheep ovaries were obtained from a local slaughterhouse and transferred to the laboratory at 37°C within 2 h from slaughter. Cumulus-oocyte complexes (COCs) were aspirated using 21G needles in the presence of 4-(2- hydroxyethyl)-1-piperazineethanesulfonic acid (HEPES)-buffered TCM-199 medium (Gibco, Life Technologies, Milan, Italy) with 0.005% (w/v) heparin. Only COCs with at least two layers of compact cumulus cells were selected for *In Vitro* Maturation (IVM). In 4-well dishes, 500 μL of IVM medium [bicarbonate-buffered TCM-199 (Gibco) containing 2 mM glutamine, 0.3 mM sodium pyruvate, 100 μM cysteamine, 10% (v/v) fetal bovine serum (FBS; Gibco), 5 μg/mL follicle-stimulating hormone (Ovagen, ICP, Auckland, New Zealand), 5 μg/mL luteinizing hormone, and 1 μg/mL 17 β-estradiol] was added to each well. Maturation was performed in a humidified atmosphere at 38.5°C and 5% CO_2_ in air for 24 h, as previously described (19). After IVM, MII (Metaphase II) oocytes with expanded cumulus and normal morphology were selected for ICSI. The cumulus cells were removed by fast pipetting of the COCs in 500 μL hepes-buffered TCM-199 with 0.4% bovine serum albumin (BSA; w:v; H199) and 300 U/mL hyaluronidase. The oocytes were washed three times in H199 and incubated in a Petri dish, pending injection.

### Intracytoplasmic sperm injection

Only oocytes with a visible first polar body were processed by Intracytoplasmic Sperm Injection (ICSI). Rehydrated spermatozoa (5-μL aliquots) were suspended in 100 μL of H199 with 0.4% BSA (w:v) and then diluted 1:1 with 12% (w:v) polyvinylpyrrolidone in PBS. Three 10–μL drops were placed on the lid of a Petri dish on a warm microscope stage (38.5°C) and covered with warm mineral oil (38.5°C). Fertilization was performed on an inverted microscope (Eclipse Ti2-U, Nikon) connected to a micromanipulation system (NT-88NEN, Narishige, Tokyo, Japan) and a piezo-driven micropipette system (PiezoXpert, Eppendorf, Milan, Italy). The oocytes were injected 24 h after the start of the IVM. The polyvinylpyrrolidone/sperm-containing drops were renewed every 10 oocyte injections. After injection, the oocytes were chemically activated by incubation in 5 μM ionomycin in H199 + 0.4% BSA for 5 min, washed once in H199 + 0.4% BSA for 5 min, and then placed in IVC-Medium (BO-IVC, cat. 71,005; ivf Bioscience, Falmouth, UK) + 10 μg/mL cycloheximide for 3.5 h in humidified atmosphere at 38.5°C and 5% CO_2_. Subsequently, they were moved to embryo culture, as described below.

### *In vitro* embryo culture

The embryo culture was performed following our standard lab procedure (16) with slight changes. Briefly, all presumptive zygotes were cultured five per 20-μL IVC-Medium (BO-IVC, cat. 71,005; ivf Bioscience) drop, covered with mineral oil (Mineral Oil, cat. 51,002; ivf Bioscience), and placed in humidified atmosphere at 38.5°C with 5% CO_2_ and 7% O_2_ for 7–8 days. The *in vitro* development was evaluated 24 h after activation for cleavage (only the 2-cell embryos were considered to have cleaved) and on days 7–8 for expanded blastocyst formation. Embryo observation and image acquisition were made on an inverted microscope (Eclipse Ti2-U; Nikon) using Octax EyeWare Imaging Software (version 2.3.0.372; Vitrolife, Västra Frölunda, Sweden).

### Pronuclear staining

To visualize pronuclei (2PN) in embryos fertilized by freeze-dried spermatozoa, a total of 28 presumptive zygotes were fixed in 4% paraformaldehyde (PFA) for 20 min, at 14–16 h after spermatozoa injection/chemical activation. Subsequently, the presumptive zygotes were permeabilized with 0.1% Triton X-100, stained with 5 μg/mL of propidium iodide (PI) for 5 min at room temperature, washed twice in 0.4% polyvinylpyrrolidone (PVP) in PBS, and then mounted on slides. Images were captured using a confocal microscope (Nikon Eclipse Ti-E).

### Statistical analysis

Data obtained from flow cytometry (DNA and acrosome integrity), *in vitro* embryo development (cleaved embryos and expanded blastocysts), and glycerophospholipids (spermatozoa fatty acids) were analyzed using SAS, v 9.4 (SAS Institute Inc., Cary, NC, USA). The general linear model evaluated the effect of the four experimental treatments on *in vitro* quality traits (sperm quality and embryo development) and the fatty acids profile. Results are presented as adjusted least squares means ± standard errors of the means.

The global lipidomic and lipidomic data were also assessed in GraphPad Prism for Windows (Version 6.01, GraphPad Software, San Diego, CA, USA) using a one-way [Kruskal–Wallis *H* test and two-way ANOVA nonparametric tests (Friedmann’s test)], respectively.

Multi-dimensional preference (MDPREF) analysis explored the *in vitro* quality traits (sperm quality and embryo development) and the fatty acid profile that correlated with the two sperm drying protocols (FD and VDE) and the two storage temperatures (RT and 4°C). It is a principal component analysis that detects linear and non-linear variable transformations using the alternating least squares method, which optimizes the transformed variables’ correlation properties or the covariance matrix. MDPREF analysis identifies the most salient variability to the preference patterns of the drying protocols and storage temperatures toward the *in vitro* quality traits (sperm quality and embryo development) and the fatty acids profile, and extracts it as the first principal component.

Statistical significance was set at *p* < 0.05.

## Results

### Effects of storage conditions and desiccation and packaging protocols on sperm quality

**Figure 2:**
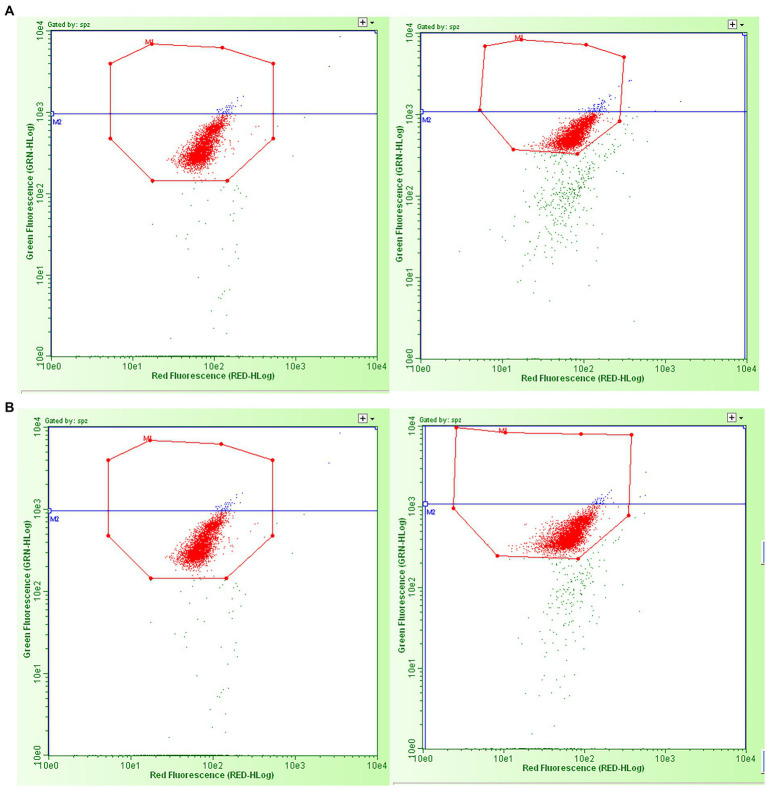
Examples of green vs. red fluorescence intensity scatter plots obtained by flow cytometric sperm chromatin structure assay analysis of the dried spermatozoa from the four experimental groups. **(A)** Left: freeze-dried and stored at room temperature; right: vacuum-dried, encapsulated, and stored at room temperature. **(B)** Left: freeze-dried and stored at 4°C; right: vacuum-dried, encapsulated, and stored at 4°C. The red dots in the octagons represent two populations of sperm cells: sperm with the double strands (with no DNA fragmentation) and sperm with single-strand DNA breaks (with “fragmented DNA” - %DFI and “high fragmented DNA - %HG). The blue dots in the octagons represent the percentage of spermatozoa with high green fluorescence, i.e., the immature cells part of DFI/cells with “high fragmented DNA.” The green dots outside the octagons are non-sperm events, considered as debris.

Samples produced by freeze-drying (FD) and vacuum-drying and encapsulation (VDE) were stored for 2 years at 4°C (4C) and room temperature (RT; Figure 1). Both drying and storage techniques preserved a high degree of DNA integrity, with DNA damage levels below the threshold for subfertility. The DNA fragmentation index (%DFI) did not undergo significant variations between the experimental groups. In the other side, the percentage of high green fluorescence (%HG) in FD_RT was lower than FD_4C (Table 1 and Figure 2).

**Table 1:** Acrosome integrity and DNA fragmentation evaluated by flow cytometry in dried-rehydrated spermatozoa.

Variable	Experimental group				
	FD_4C	FD_RT	VDE_4C	VDE_RT	EG effect
DI (%)	17.95 ± 5.35	32.73 ± 6.55	24.68 ± 5.35	28.67 ± 5.35	ns
DR (%)	82.05 ± 5.34	67.27 ± 6.54	75.33 ± 5.34	71.33 ± 5.34	ns
% DFI	1.103 ± 0.099	0.860 ± 0.121	1.033 ± 0.099	1.108 ± 0.099	ns
% HG	0.748 ± 0.135^ab^	0.520 ± 0.166^a^	1.060 ± 0.135^b^	0.768 ± 0.135^ab^	*P* < 0.05

Values are presented as least square means and standard error of the means. Different superscript letters in the same row correspond to a significant (*P* < 0.05) difference between experimental groups, “ns” means not significant. EG, experimental group; FD, freeze-drying; VDE, vacuum-dried and encapsulated; 4C, storage at 4°C; RT, storage at room temperature; DI, dead spermatozoa with intact acrosome; DR, dead spermatozoa with damaged acrosome; %DFI, DNA fragmentation index; %HG, sperm with high green fluorescence, describes poor chromatin condensation.

**Figure 3:**
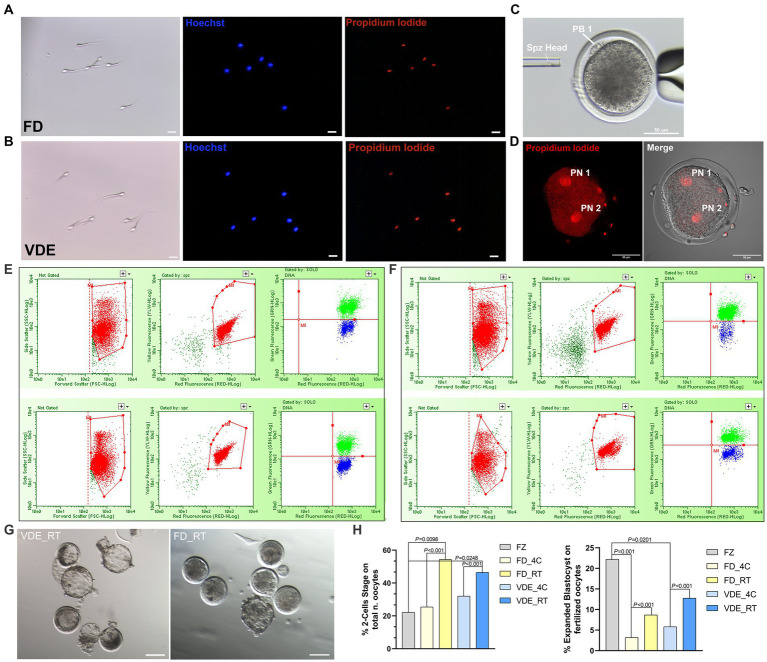
Post-rehydration sperm morphology and embryonic development. **(A)** Freeze-dried (FD) and **(B)** vacuum-dried and encapsulated (VDE) spermatozoa stained with Hoechst 33342 (blue signal) and propidium iodide (red signal) after rehydration. All cells were positive for propidium iodide. Scale bar = 20 μm. **(C)** Intracytoplasmic sperm injection of a dried-rehydrated spermatozoon into an *in vitro* matured sheep oocyte. Spz Head, sperm head; PB1, first polar body. Scale bar = 50 μm. **(D)** Activated oocyte fertilized by freeze-dried spermatozoa via ICSI with two distinguishable pronuclei: Pronuclear 1 (PN 1) and Pronuclear 2 (PN 2), Scale bar = 50 μm. Flow cytometric analysis of acrosome integrity in FD **(E)** and VDE **(F)** spermatozoa. The red population represent: in the first two panels, the total events acquired; in the second two panels, the events of the first panel plotted in the second panel considering only sperm cells, excluding debris. The green and blue populations represent dead spermatozoa with damaged and intact acrosomes, respectively. The top row in **(E,F)** shows an example of spermatozoa analyzed after storage for 2 years at 4°C; the bottom row shows the respective results of spermatozoa stored for 2 years at room temperature. **(G)** Blastocysts produced via ICSI of VDE (on the left) and FD (on the right) spermatozoa stored for 2 years at room temperature (RT) at day 7th of *in vitro* embryo culture. Scale bar = 100 μm. **(H)** *In vitro* embryo development outcomes, 2-Cells Stage embryos (on the left) and expanded blastocyst (on the right). FZ, frozen/thawed spermatozoa.

The cellular membrane of all spermatozoa in both desiccation methods and storage types was damaged after rehydration, as confirmed in the total positivity to PI (Figures 3A,B).

Post-rehydration structural assessment of intact acrosome (DI%) were statistically insignificant, anyway the FD_RT group showed the highest proportion of acrosome integrity (Figures 3E,F and Table 1).

### Vacuum-dry encapsulation better preserve sperm fertilizing ability after ICSI in RT storage

As an initial assessment to rule out the development of haploid parthenogenic embryos arising from the chemical activation of oocytes fertilized via ICSI (Figure 3C), was examined the rate of 2-pronuclei (2PN) embryo formation. As illustrated in Figure 3D, oocytes that were fertilized using freeze-dried spermatozoa and subsequently subjected to chemical activation involving ionomycin and cycloheximide exhibited a 2PN formation rate of 78.57% (22/28). Rehydrated spermatozoa injected into sheep *in vitro*-matured oocytes (Figure 3C) led to a higher embryo cleavage when stored at RT than 4°C in the FD [54.3% (*n* = 62) vs. 25.4% (*n* = 83); *p* < 0.001] and VDE [46.5% (*n* = 53) vs. 32.0% (*n* = 70); *p* < 0.001; Figure 3H] groups. Additionally, when utilizing frozen/thawed semen (FZ), there was a reduced formation of 2-cell embryos (FZ, 22.22%, *n* = 36) in comparison to FD_RT (*p* = 0.0098) and VDE_RT (*p* = 0.0248). RT also had a higher day-8 expanded blastocyst formation rate than 4°C in the FD (8.7% vs. 3.2%; *p* < 0.001) and, even more apparent, VDE (12.8% vs. 5.8%; *p* < 0.001; Figures 3G,H) groups. Furthermore, in the comparison with the FZ group, there was a greater formation of expanded blastocysts (FZ, 22.22%, *n* = 36) compared to FD_4C (*p* = 0.001) and VDE_4C (*p* = 0.0201).

### Dynamics of glycerophospholipid composition in FD and VDE ram spermatozoa

Table 2 provides a selection of the global lipidomic analysis. The entire list of 29 fatty acid can be found as Supplementary Table S1. Docosapentaenoic acid (DPA; C 22:5 ω3) in the FD_RT group was higher than in the FD_4C group (0.70 ± 0.04 vs. 0.55 ± 0.04) and lower in the VDE_RT group than in the VDE_4C group (0.54 ± 0.04 vs. 0.67 ± 0.04). Docosahexaenoic acid (DHA; C 22:6 ω3) in both VDE storage groups was significantly higher than in the respective FD samples. Moreover, all dry semen samples (FD_4C, FD_RT, VDE_4C, and VDE_RT) exhibited a significant elevation in DHA levels when compared to fresh semen. Spermatozoa in the VDE_RT group presented a higher quantity of monounsaturated fatty acids (MUFA; 10.91 ± 0.47) and omega-6 polyunsaturated fatty acids (PUFA ω-6; 11.26 ± 0.23) than all other groups. The VDE_4C group had significantly higher omega-3 PUFA (PUFA ω-3) than the VDE_RT group (59.14 ± 1.72 vs. 53.06 ± 1.72, *p* < 0.05). The total PUFA quantity (PUFA TOT) in the VDE_RT samples was higher than in the FD_RT samples.

**Table 2:** Dried-rehydrates sperm fatty acid analysis in the four experimental groups.

Sperm fatty acids	Experimental group (Values are presented as a % of the total fatty acids)				
	FRESH	FD_4C	FD_RT	VDE_4C	VDE_RT
Docosapentaenoic acid (DPA) C22:5n3	0.67 ± 0.17	0.55 ± 0.04	0.70 ± 0.04	0.67 ± 0.04	0.54 ± 0.04
Docosahexaenoic Acid (DHA) C22:6n3	5.53 ± 0.78	46.77 ± 2.83^#†^	47.47 ± 2.83^#†^	57.13 ± 2.83^#†^	51.08 ± 2.83^#†^
Saturated fatty acids (SFA)^a^	30.66 ± 1.65	35.26 ± 1.38	35.39 ± 1.38	27.86 ± 1.38	24.72 ± 1.38
Monounsaturated Fatty acids (MUFA)^b^	13.25 ± 1.30	4.32 ± 0.47	4.48 ± 0.47^#^	3.65 ± 0.47*^†^	10.91 ± 0.47*^#^
Omega-6 fatty Acids (PUFAω6)^c^	5.77 ± 0.39	9.4 ± 0.23^†^	9.07 ± 0.23^#†^	9.31 ± 0.23*^†^	11.26 ± 0.23*^#†^
Omega-3 fatty Acids (PUFAω3)^d^	48.98 ± 3.12	50.85 ± 1.72	50.99 ± 1.72^#^	59.14 ± 1.72*	53.06 ± 1.72*^#^
All polyunsaturated fatty acids (PUFATOT)^e^	54.73 ± 2.78	60.24 ± 1.83	60.05 ± 1.83^#^	68.45 ± 1.83	64.31 ± 1.83^#^
All trans fatty acids (TRANSTOT)^f^	1.36 ± 0.10	0.19 ± 0.08	0.10 ± 0.08^†^	0.06 ± 0.08^†^	0.08 ± 0.08^†^

FD, freeze-drying; VDE, vacuum-dried and encapsulated; 4C, storage at 4°C; RT, storage at room temperature. ^a^SFA: C14:0 + C16:0 + C17:0 + C18:0 + C20:0 + C21:0 + C22:0 + C24:0. ^b^MUFA: C16:1 9cis + C18:1 9 cis + C18:1 11cis + C22:1 cis + C24:1 cis. ^c^PUFAω6: C18:2 ω6 + C18:3 ω6 + C20:2 ω6 + C20:3 ω6 + C20:4 ω6. ^d^PUFAω3: C18:3 ω3 + C20:3 cisω3 + C20:5 ω3 + C22:5 ω3 + C22:6 ω3. ^e^PUFATOT: PUFAω6+ PUFAω3. ^f^TRANSTOT: C18:1 trans 9. ^*^In the same row correspond to a significant difference between storage temperatures within the same drying technique; ^#^in the same row correspond to a significant difference between drying techniques within the same storage temperature; ^†^in the same row correspond to a significant difference between FRESH group and all remaining groups. Statistical significance was set at *p* < 0.05.

### Freeze-drying and vacuum-drying with encapsulation shape the polyunsaturated fatty acid distribution

The changes in membrane fatty acid composition of frozen/thawed ram spermatozoa were well documented (20); likewise, this work reports on similar changes in the FD and VDE spermatozoa.

**Figure 4:**
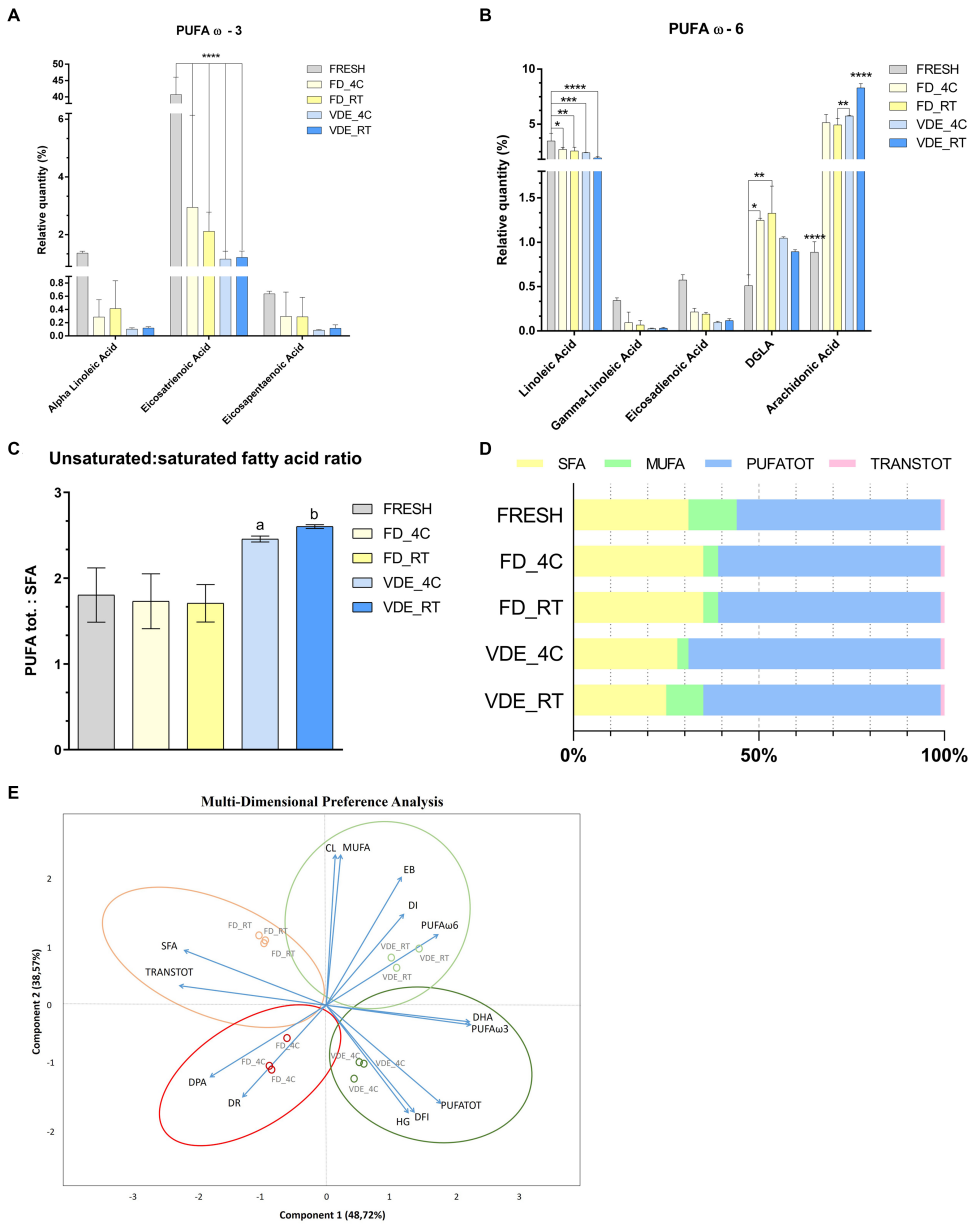
Polyunsaturated fatty acid (PUFA) profile and multi-dimensional preference analysis biplot. **(A)** PUFA ω3; **** indicates *p* < 0.001. **(B)** PUFA ω-6; **** indicates *p* < 0.001; *, **, and *** indicate *p* < 0.05. **(C)** PUFA tot. to saturated fatty acid (SFA) ratio. “a” indicates VDE_4C vs. FRESH (*p* = 0.0254), VDE_4C vs. FD_4C (*p* = 0.0161), VDE_4C vs. FD_RT (*p* = 0.0152). “b” indicates VDE_RT vs. FRESH (*p* = 0.0114), VDE_RT vs. FD_4C (*p* = 0.070), VDE_RT vs. FD_RT (*p* = 0.0064). **(D)** Proportion as a percentage of the total fat\ty acids of SFA, MUFA, PUFATOT and TRANSTOT. **(E)** Multi-dimensional preference analysis biplot based on a combination of *in vitro* quality parameters, including sperm quality [acrosome integrity (dead-intact, DI, or dead-reacted, DR) and chromatin stability (DNA fragmentation index, %DFI, and high green fluorescence, %HG)], embryo development [cleaved (CL) and expanded blastocyst (EB)], fatty acid profile (DPA, DHA, SFA, MUFA, PUFA ω-6, PUFA ω-3, PUFATOT, TRANSTOT), and the four experimental groups (FD_4C, FD_RT, VDE_4C, VDE_RT). The dots indicate the exact positions of samples in the four experimental groups between the two components. The ellipses and circle represent clustering based on the *in vitro* quality parameters (sperm quality, embryo development, and fatty acid profile). SFA, all saturated fatty acids combined; DPA, docosapentaenoic acid; DHA, docosahexaenoic acid; MUFA, mono-unsaturated fatty acids; PUFATOT (or PUFA TOT), all polyunsaturated fatty acids combined; TRANSTOT, all trans fatty acids combined; FRESH, refrigerated spermatozoa; FD_4C, freeze-dried and stored at 4°C; FD_RT, freeze-dried and stored at room temperature; VDE_4C, vacuum-died, encapsulated, and stored at 4°C; VDE_RT, vacuum-dried, encapsulated, and stored at room temperature.

The most dramatic changes were recorded for C20:3 cis ω-3 (Eicosatrienoic acid). It significantly (*p* < 0.001; Figure 4A) decreased from 40.74 ± 5.32 in the fresh samples to 2.95 ± 3.19 in FD_4C, 2.12 ± 0.67 in FD_4C, 1.16 ± 0.27 in VDE_4C, and 1.21 ± 0.22 in VDE_RT. A similar but less distinct trend was noted for C18:2 ω-6 (linoleic acid), decreasing from 3.46 ± 0.71 to the following respective values: 2.70 ± 0.18, 2.55 ± 0.35, 2.40 ± 0.03, and 1.93 ± 0.11; *p* < 0.05; Figure 4B). The C20:3 ω-6 (dihomo-γ-linolenic acid) in FD_RT (1.33 ± 0.31) was higher than in FD_4C (1.25 ± 0.03) and fresh (0.51 ± 0.13). The C20:4 ω-6 (arachidonic acid) level in VDE_RT (8.28 ± 0.40) was higher than in fresh (0.89 ± 0.120, FD_4C (5.13 ± 0.75), FD_RT (4.93 ± 0.59), and VDE_4C (5.73 ± 0.07; *p* < 0.001). Furthermore, the arachidonic acid level in the fresh samples was lower than in all FD and VDE groups (*p* < 0.001), and that in FR_RT lower than in VDE_4C (*p* = 0.010).

The ratio of PUFA to saturated fatty acids (SFA) revealed that the VDE_4C (2.46 ± 0.02) and VDE_RT (2.60 ± 0.01) groups exhibited higher values compared to the remaining groups (Fresh: 1.80 ± 0.18; FD_4C: 1.73 ± 0.19; FD_RT: 1.71 ± 0.13; *p* < 0.05; Figures 4C, D).

### Possible association between *in vitro* quality features, fatty acids, desiccation method, and storage conditions

Multi-dimensional preference (MDPREF; Figure 4E) analysis showed that most variability was due to the first two components (∼87%). The analysis produced four dimensions based on the four experimental groups (FD_4C, FD_RT, VDE_4C, and VDE_RT), with their relative sperm quality variables’ associations.

Among the experimental groups, a positive relationship between the DNA quality variables (%DFI and %HG) and VDE_4C was noted, while FD-RT showed the opposite trend. The embryo development traits (cleavage and expanded blastocyst) and good-quality sperm membrane (intact acrosome) were positively associated with VDE_RT and negatively associated with FD_4C, in which the sperm membrane quality was poor (reacted acrosome) and contained a lower DPA level. A positive association was observed between MUFA and PUFA (ω-3 and DHA, ω-6, and PUFA TOT) and the VDE samples. A good association was noted between SFA and TRANS TOT fatty acid and the FD_RT samples.

## Discussion

The first successful fertility preservation of dried spermatozoa has been achieved using lyophilization (21, 22); therefore, this method became the default water extraction strategy in almost all the subsequent studies. This study describes and analyzes a previously unexplored drying technique, vacuum drying, which has been found to be less invasive for the structural integrity of spermatozoa glycerophospholipids, acrosome, and DNA levels. Moreover, this method has demonstrated improved fertilization outcomes when evaluated in pre-implantation stage embryos.

The benefits of this new sperm drying method were tracked even after 2 years of storage at 4°C and RT; remarkedly and unexpectedly, the latter storing approach proved the most appropriate (Table 1). As previously noted, the data presents an entirely unexpected outcome. However, it is conceivable that an explanation for this phenomenon can be found in the nature of the membrane lipids of ram spermatozoa. These lipids may create more favorable conditions for preservation at RT in anhydrous state, as elaborated upon in the following discussion.

Sperm lipid changes during cryopreservation have been extensively investigated (23). Collectively, the published data indicate susceptibility to cold shock and differences among species in lipid phase transition and sperm survival that seem to be linked to their membranes’ PUFA (polyunsaturated fatty acids) to SFA (saturated fatty acids) ratio (24) and cholesterol content (25). However, little is known about lipid changes during sperm drying. To fill this knowledge gap, we analyzed lipid composition and its variations following various drying and storage conditions in ram spermatozoa. Ram spermatozoa, like those of bull (26) and boar (27), are characterized by a high PUFA to SFA ratio (28). Our fresh ram spermatozoa data presented a PUFA to SFA (TOT) ratio of roughly 60:35 (1.71), in agreement with these previous reports. The different proportions of MUFA, PUFA, and SFA have a significant effect on the physical and chemical properties of the membrane, including its fluidity at room temperature (29) and lipid phase transition temperature (30). The lipid conformation of ejaculated ram spermatozoa makes them highly susceptible to cold shock (27). This high susceptibility could partially explain the membrane damage and acrosomal loss in our semen samples as they were stored at 4°C during transport to France or frozen at a slow cooling rate (1°C per minute) before water sublimation in the FD groups. It is possible that storage of the dried samples at 4°C for 2 years slowly but progressively enhanced the damage to the membranes. In any case, it’s worth noting that post-rehydration, freeze-dried sperm display complete immobility (a condition significantly distinct from that of frozen semen). This observation underscores the impact of lyophilization, likely connected to the inherent nature of the technique, on the integrity of the sperm’s outer membrane.

Functional tests using ICSI fertilization indicated a yield of semen stored at 4°C comparable to that stored at RT.

Long-term sperm storage at RT was reported in mice (31) and rabbits (32). However, the length of RT storage in those studies was shorter, a maximum of 1 year (33), than the duration reported in our work. Our storage conditions were intentionally kept straightforward, involving the placement of capsules/vials in a drawer. Nevertheless, it’s worth noting that the office environment is carefully controlled, with a temperature ranging between 20°C and 25°C and a humidity level maintained between 40 and 60% (Figure 1E).

It is well-recognized that the use of chelating agents such as EGTA and EDTA has several protective functions against DNA damage in lyophilized spermatozoa (34). It is well corroborated that the physical processes during lyophilization (freezing and vacuum-drying) can induce DNA breaks (7). Membrane breaks lead to the release of divalent cations that activate endonucleases such as DNase and promote DNA breaks; adding chelating agents to the lyophilization medium could suppress their activity (35). The alkaline pH and EGTA in our lyophilization medium could suppress such DNAse activity (36). We have previously shown how freeze-dried ram spermatozoa can maintain DNA integrity, including EGTA in the lyophilization media (10). This could explain why DNA integrity was maintained in this experimental design, even though the spermatozoa were dried by lyophilization and VDE and stored at RT. Nevertheless, the significant disparity in %HG (high green fluorescence cells), indicative of reduced chromatin condensation, could potentially signify an epigenetic influence on embryos and, perhaps even more notably, on the offspring of large mammals. This aspect, which has not been assessed in this study, demands heightened attention. Especially concerning the welfare of these animals, it is imperative to give substantial consideration to the possibility of ensuring the birth of entirely healthy offspring from freeze-dried spermatozoa, a prudent course of action when dealing with large mammals.

Spermatozoa processed in VDE maintained better fertilizing capacity than the FD ones stored under vacuum, even when kept at RT (Figure 3H). Presumably, the argon (90%) and helium (10%) inside the stainless steel capsules (15, 37, 38) prevented the structural decay in the VDE samples caused by oxidative damage. Our DNA integrity, fertilization capacity, and lipidomic findings jointly support this assertion.

This study was the first to present a lipidomic map of ram spermatozoa that tracked the changes following various dehydration techniques and storage conditions and associated these with membrane integrity, DNA damage, and embryo development. Perhaps the most relevant information gained through this lipid analysis was the high proportion of DHA (40%) in all dry samples in comparison to the control (fresh semen: 5.5%). DHA is highly sensitive to oxidation, particularly at low temperatures (39), explaining why dried spermatozoa packaged in a saturated inert gas atmosphere were preserved better than under incomplete vacuum, as reported in our work. The PUFA TOT value in spermatozoa dried by the VDE technique and stored at RT was higher than in the respective FD ones. Our analysis showed a strong association between PUFA ω-3 and storage at 4°C and between PUFA ω-6 and storage at RT in the VDE samples.

The changes detected by the lipid analysis in the FD and VDE spermatozoa can be put in context with the structural and functional parameters and storage conditions assessed in this study by applying the MDPREF analysis biplot. The most significative association gleaned from the MDPREF analysis plot is the positive association between high MUFA and PUFA ω-6 percentages, structural and DNA integrity, and embryonic development in the VDE samples stored at RT (Figure 4E). Conversely, long-term storage at 4°C appeared positively associated with DNA fragmentation, acrosome loss, and lipid peroxidation. A trait highlighted by MDPREF analysis in all samples was the strong correlation between PUFA TOT and DNA damage, corroborating with Lewis (40), who revealed that spermatozoa were susceptible to lipid peroxidation-mediated DNA damage in the presence of a high proportion of PUFA. Our study also found a positive relationship between DPA and disrupted acrosome rate. The same condition was revealed in mice, where a decrease in DPA was associated with impaired acrosome reaction, resulting in negative consequences for fertilization (41).

High PUFA levels (~50%) were reportedly positively correlated with intact membranes in post-thaw mouse spermatozoa (42). Our findings suggest that the higher levels of DHA, MUFA, PUFA beneventofrosinoneω-3, and PUFA ω-6 detected in the VDE stored at RT than FD samples were presumably involved in their better structural and functional performances.

While ICSI remains the sole applicable method for freeze-dried semen due to the current lack of motility in post-rehydrated spermatozoa, it still cannot be deemed entirely eco-friendly. This is because it necessitates the use of microscopes, CO_2_, disposable plastics, and various other equipment and resources. This stands in stark contrast to the practices of *in vivo* fertilization in animal husbandry, which relies on refrigerated or frozen semen.

Nevertheless, it is worth noting that freeze-drying, in and of itself, has the potential to significantly reduce the environmental impact associated with the use of cryogenic fluids like liquid nitrogen. This inherently offers substantial energy savings and eliminates pollution to a great extent. So far, this alternative preservation method is particularly well-suited for use with wild animals, where the limitations and challenges of using liquid nitrogen are considerable.

In conclusion, this work demonstrated that an alternative drying solution for lyophilization, vacuum-drying with encapsulation, resulted in enhanced preservation of the structural and fertilization potential of ram spermatozoa stored for 2 years both at room temperature and 4°C. Furthermore, from the embryonic development data it can be seen that the storage yield of samples stored at RT is comparable with those stored at 4°C, especially in the VDE method. The explanation could arise from the VDE packaging, an inert atmosphere and a perfectly sealed capsule could allow a potential totally green application of the freeze-dried spermatozoa. Furthermore, the lipidomics data show a strong change in the composition of fatty acids in relation to the freeze-drying method used. Different species have different sperm lipid mapping, so it would be advisable to choose a more compliant freeze-drying method depending on the species of interest. Nevertheless, it is essential to acknowledge that we can only determine the optimal freeze-drying and preservation conditions for ram spermatozoa once we have successfully achieved the birth of the first offspring using freeze-dried sperm. This milestone will serve as a crucial reference point for guiding future research in this field.

## Data availability statement

The original contributions presented in the study are included in the article/Supplementary material, further inquiries can be directed to the corresponding authors.

## Ethics statement

The animal study was approved by Italian Ministry of Health (No. 200/2017-PR, Prot. 944F0.1 del 04/11/2016). The study was conducted in accordance with the local legislation and institutional requirements.

## Author contributions

LP: Conceptualization, Writing – original draft, Data curation, Formal analysis, Investigation, Methodology, Writing – review & editing. FT: Conceptualization, Data curation, Formal analysis, Investigation, Methodology, Validation, Writing – original draft. DA: Methodology, Writing – review & editing. JS: Writing – review & editing. JB: Writing – review & editing. MCo: Writing – review & editing, Methodology. ST: Writing – review & editing, Methodology. FP: Writing – review & editing. AL: Writing – review & editing. KM: Writing – review & editing, Methodology. MCz: Writing – review & editing, Data curation, Funding acquisition. PL: Writing – review & editing, Data curation, Funding acquisition, Conceptualization, Project administration, Resources, Supervision, Writing – original draft.
